# Relationships between external loads, sRPE-load, and self-reported soreness across a men’s collegiate soccer season

**DOI:** 10.5114/biolsport.2023.125587

**Published:** 2023-04-06

**Authors:** Nicholas M. Kuhlman, Margaret T. Jones, Andrew R. Jagim, Mary Kate Feit, Richard Aziz, Thomas Crabill, Jennifer B. Fields

**Affiliations:** 1 Exercise Science and Athletic Training, Springfield College, Springfield, MA; 2 Patriot Performance Laboratory, Frank Pettrone Center for Sports Performance, George Mason University, Fairfax, VA; 3 Sport, Recreation, and Tourism Management, George Mason University, Fairfax, VA; 4 Sports Medicine Department, Mayo Clinic Health System, La Crosse, WI; 5 Physical Education and Health Education, Springfield College, Springfield, MA

**Keywords:** Athletes, GPS, Soreness, Workloads, Internal load

## Abstract

The purpose was to examine relationships between external loads (ELs), perceived exertion, and soreness. Collegiate men soccer players (n = 19) were monitored for 72 sessions (training: n = 53; matches: n = 19). Likert scale assessments (0–6) of lower body soreness were collected prior to each session, and ELs were collected using positional monitoring technology. Session rate of perceived exertion (sRPE-load) was calculated by multiplying perceived exertion values (Borg CR-10 Scale) by respective session duration to determine internal load. Multiple analyses of variance were used to determine differences in ELs across seasons (pre-season, in-season, post-season) and sessions (training, match). Bivariate Pearson correlation coefficients and linear regression analyses were used to evaluate relationships among soreness, ELs, and sRPE-load. Greatest ELs were observed during pre-season and post-season phases (p < 0.001). Sessions with high perceived exertion and low soreness were associated with higher ELs (p < 0.05). Duration (t = 16.13), total distance (t = 9.17), sprint distance (t = 7.54), player load (t = 4.22), top speed (t = 4.69), and acceleration (t = 2.02) positively predicted sRPE-load (F = 412.9, p < 0.001, R**^2^** = 0.75). Soreness was weakly and trivially correlated with ELs (p < 0.05). The very strong relationship between ELs and sRPE-load highlights the utility of sRPE-load as a practical means to estimate workload; however, more research into the relationship between soreness and workload is warranted.

## INTRODUCTION

Preparation for soccer is often characterized by periodization schemes that target technical, tactical, and physical development throughout the year to meet competition demands and mitigate injury risks [1–3]. This process involves strategic manipulation of training variables (i.e., volume, intensity, frequency, density) at key time points to elicit sport-specific adaptations [1, 4]. However, it can be challenging to quantify sport-specific training demands outside of resistance training and conditioning-related activities. Further, within the current National Collegiate Athletic Association (NCAA) format, constraints exist that may hinder optimal preparation and performance. For example, a two-week pre-season precedes a congested competitive season, in which athletes may compete in > 20 matches over 12–15 weeks [1, 5]. This season-structure underscores the importance of quantifying training in a manner that is practical while sensitive to the potential risks associated with a congested season, such as non-functional overreaching, overtraining syndrome, illness, and injury [1, 6–9].

Technical and tactical components of soccer training are often designed with prescriptions of external load (EL) parameters, which include volume and intensity metrics. The use of wearable technologies, such as global positioning systems (GPS) equipped with inertial sensors, enables coaches to quantify ELs while reducing error and guesswork in a streamlined process [6, 10]. Despite its cost-prohibitive nature, GPS implementation creates opportunities for real-time observation of EL metrics as well as a retrospective analysis of broader trends across different season phases. Previous studies have reported higher ELs during pre-season compared to in-season for various professional [11] and collegiate [12] soccer populations. These observations are likely the result of an increased focus on rebuilding physical capacities, compounded by a short pre-season, leading to an intensified training period prior to the start of the competitive season [12]. Differences between in-season and post-season phases have been investigated in DI soccer players, with reductions observed in post-season ELs compared to in-season [12]. These findings may reflect either the intentional reduction of EL prescription during post-season training in order to elicit a “peaking” effect or, by contrast, the chronic accumulation of fatigue leading to attenuated post-season physical outputs [12, 13]. However, further investigation is necessary to determine differences between in- and post-season ELs in DIII soccer players.

Despite the benefits of GPS monitoring, EL is likely not the sole driver of physiological adaptation. Rather, internal load (IL) – the physiological stress imposed by the completion of EL – is thought to mediate the adaptive responses from training [6, 14]. Moreover, in team sports like soccer, a given EL can elicit significant variation in IL between players [6]. Thus, IL metrics can be useful in quantifying team and individual responses to training. One cost-effective and valid method of estimating IL is the session Rating of Perceived Exertion (sRPE-load) [15], which is strongly correlated with EL in a variety of athletic populations [6, 16–18] and may serve as an effective proxy for EL. Thus, a relationship between external and internal loads seems to exist, but because of large differences that occur in the physical demands of athletes across levels of play and sports, continued investigation is warranted.

However, EL and sRPE-load measures alone may not represent the totality of physical stress incurred nor do they provide insight into the recovery status of athletes over successive days, weeks, and months of training and competition. Therefore, other IL indices, such as measures of physical wellness, may be used to inform training prescription and track recovery status [19]. Customized self-reported perceived wellness measures have garnered interest as time-efficient, cost-effective, and valid measures of IL to quantify and monitor more holistic trends in the constructs of fatigue, recovery, muscle soreness, and stress [20–22]. While previous investigations have observed correlations between perceived muscle soreness and EL across two-week time periods [21, 23], a longitudinal examination of these associations across a full collegiate soccer season has not been conducted. Thus, the purpose of the current study was to quantify ELs and examine the relationships between sRPE-load, and perceived soreness in training sessions across a collegiate men’s soccer season.

## MATERIALS AND METHODS

### Subjects

National Collegiate Athletic Association (NCAA) Division III men soccer athletes (n = 19) classified as “starters” (i.e., those players that participated in > 50% of a match’s duration) participated [12, 24, 25]. Goalkeepers and non-starters were excluded due to the relatively low total distances they traveled. All athletes were under the direction of a Certified Strength and Conditioning Specialist**^®^** and were following similar training regimens. All athletes completed a medical history form and were cleared for intercollegiate athletic participation. Risks and benefits were explained to athletes, and an institutionally approved written informed consent form was signed before participation. All procedures involving human subjects were conducted in accordance with the requirements of the Declaration of Helsinki and approved by the college’s Institutional Review Board for Human Subjects (IRB # 3182021).

### Study Design

Data were collected over 12 weeks during the 2021 NCAA men’s soccer season (training sessions: n = 53; matches: n = 19 [2 scrimmages, 17 competitions]) for a total of 1,368 individual player sessions (Table 1). Lower body soreness was collected each afternoon, prior to training, while athlete RPE was collected approximately 15 minutes after the cessation of each training session. Lower body soreness and RPE measures were collected on training days only; match days were excluded. EL was quantified during all field training sessions and matches using 10 Hz GPS/GNSS technology (Player Tek, Catapult Sports, Melbourne, Australia). The 10 Hz units have been shown to provide a valid and reliable estimate of kinematic data with sufficient inter-unit reliability for comparisons between athletes [26, 27]. These devices use a minimum of 3 satellites, and units were turned on outside 30 minutes before sessions. To promote reliability, players wore the same unit for each match throughout the season. Devices were worn according to manufacturer guidelines in a supportive harness positioned between the scapulae.

**TABLE 1:** Structure of the 12-week season

Week	Season Phase	*#* of Training Sessions	*#* of Matches	Total *#* of Sessions
1	Pre-Season	8	1*	9
2		6	1*	8
	In-Season	0	1	
3		4	2	6
4		4	2	6
5		4	2	6
6		4	2	6
7		4	1	5
8		4	1	5
9		4	2	6
10		4	2	6
11	Post-Season	3	1	4
12		4	1	5
**Total**		53	19	72

* Denotes pre-season scrimmage

### Lower Extremity Soreness

Athletes provided subjective ratings of lower body soreness (quadriceps, hamstrings) on Likert scales ranging from 0–6 (0 = complete absence of pain; 1 = light pain felt only when touched/a vague ache; 2 = moderate pain felt only when touched/a slight persistent pain; 3 = light pain when walking up or down stairs; 4 = light pain when walking on a flat surface/painful; 5 = moderate pain, stiffness, or weakness when walking/very painful; and 6 = severe pain that limits my ability to move) [28, 29]. Soreness measures were collected immediately before training sessions only; match days were excluded. To investigate the relationship between soreness and EL, sessions were stratified into high (≥ 3) and low (≤ 2) soreness classifications.

### External Load

EL metrics collected for training sessions and matches were: total distance, sprint efforts, sprint distance (> 5.5 m/s), top speed, acceleration efforts (> 3 m/s**^2^**), player load (PL), player load per minute (PL/min), and power plays (power output > 20 watts/kg > 1 sec). PL was yielded from the triaxial accelerometer within the device using a proprietary formula:

∑√(instantaneousrateofchangeinaccelerationinall3orthogonalplanes)

After each training session and match, data were downloaded using the proprietary software, which automatically detects and removes outlier data [30].

### Session Rate of Perceived Exertion

RPE was collected using the modified Borg CR-10 scale [31] for training sessions only. Athletes provided their RPE approximately 15 minutes post-training session to avoid the recency effect and to ensure their perceived intensity would reflect the entire training session. Further, each athlete reported RPE in isolation to avoid influence from teammates. Session RPE (sRPE-load) was calculated by multiplying the given RPE by the duration of the training session in minutes and expressed using arbitrary units (AU). To investigate the relationship between sRPE-load and other measures of load, sessions were stratified by RPE into high (RPE ≥ 6) and low (RPE ≤ 5) difficulty. Specifically, this approach was used to determine if differences in external workloads existed when sessions were categorized into “high” and “low” RPE [6].

### Statistical Analysis

SPSS version 25.0 (IBM, Armonk, NY) was used for summary statistics. All values are presented as *means* ± *SDs*. A multiple analysis of variance (MANOVA) was used to determine 1) seasonal differences (pre-season vs in-season vs post-season) in EL measures; 2) differences in weekly ELs between training sessions and matches; and 3) differences in ELs stratified by high and low RPE and lower body soreness. Bonferroni post hoc comparisons were calculated when a significant main effect or interaction was identified. Partial eta**^2^** (η**^2^**) effect sizes were calculated and interpreted as follows: small: 0.01–0.06; moderate: 0.06–0.14; and large: > 0.14 [32]. Bivariate Pearson and Spearman correlation coefficients determined relationships among sRPE-load, ELs, and quadricep and hamstring soreness. The strength of correlation coefficients was classified as trivial (|r| < 0.10), weak (0.10 ≤ |r| < 0.30), moderate (0.30 ≤ |r| < 0.50), strong (0.50 ≤ |r| < 0.70), very strong (0.70 ≤ |r| < 0.90), and nearly perfect (|r| ≥ 0.90) [33]. When strong-to-very strong correlations existed, a multiple linear regression was used to assess the predictive ability of EL markers on perceived measures (p < 0.05).

## RESULTS

Differences in EL and IL across the season are displayed in Table 2. Significant differences across pre-, in-, and post-season were observed in training sessions for sRPE-load, and in all sessions (training sessions and matches) for total distance, acceleration efforts, PL, PL/min, and power plays. Specifically, sRPE-load (F = 69.06, η**^2^** = 0.15), total distance (F = 47.49, η**^2^** = 0.10), and PL (F = 34.16, η**^2^** = 0.07) were highest in pre-season (sRPE-load: 480.7 ± 231 AU, 95% CI [453.3, 508.1]; total distance: 5885 ± 2065 m, 95% CI [5620, 6151]; PL: 269.9 ± 92.3 AU, 95% CI [257.9, 282.3]) compared to in-season (sRPE-load: 292.4 ± 171 AU, 95% CI [276.9, 307.9]; total distance: 4393 ± 1681 m, 95% CI [4242, 4543]; PL: 212.5 ± 78.5 AU, 95% CI [205.6, 219.3]) and post-season (sRPE-load: 326.9 ± 157 AU, 95% CI [290.81, 363.12]; total distance: 5068 ± 1809 m, 95% CI [4718, 5418]; PL: 240.6 ± 77.9 AU, 95% CI [224.6, 256.6]) (p < 0.001). Accelerations (F = 22.57, p < 0.001, η**^2^** = 0.05), PL/min (F = 18.98, p < 0.001, η**^2^** = 0.04), and power plays (F = 24.12, p < 0.001, η**^2^** = 0.06) did not differ across pre-season (accelerations: 49.1 ± 25.1, 95% CI [45.7, 52.5]; PL/min: 2.49 ± 0.51 AU, 95% CI [2.4, 2.6]; power plays: 26.4 ± 17.7, 95% CI [24.4, 28.5]) and post-season (accelerations: 43.9 ± 24.9, 95% CI [39.5, 48.4]; PL/min: 2.75 ± 0.50 AU, 95% CI [2.7, 2.8]; power plays: 23.6 ± 12.6, 95% CI [20.9, 26.2]), but were higher than their respective in-season (accelerations: 36.3 ± 21.7, 95% CI [34.3, 38.2]; PL/min: 2.76 ± 0.50 AU, 95% CI [2.7, 2.8]; power plays: 18.5 ± 12.4, 95% CI [17.4, 19.7]) values.

**TABLE 2:** Measures of athlete (n = 19) workload across the competitive season

	Pre-Season (n = 16)	In-Season (n = 47)	Post-Season (n = 9)	p-value	η2
sRPE-load (AU)*	480.7 ± 231	292.4 ± 171	326.9 ± 157	< 0.001	0.15
**Total distance (m)**	5885 ± 2065	4393 ± 1681	5068 ± 1809	< 0.001	0.10
**Sprint distance (m)**	74.4 ± 75.6	65.2 ± 86.2	74.8 ± 68.7	0.31	0.003
**Sprint efforts (#)**	1.94 ± 2.4	1.55 ± 2.2	1.88 ± 1.9	0.07	0.01
**Acceleration efforts (#)**	49.1 ± 25.1	36.3 ± 21.7	43.9 ± 24.9	< 0.001	0.05
**Top speed (m/s)**	7.22 ± 1.2	7.13 ± 1.4	7.48 ± 1.2	0.04	0.01
**Player load (AU)**	269.9 ± 92.3	212.5 ± 78.5	240.6 ± 77.9	< 0.001	0.07
**PL/min (AU/min)**	2.49 ± 0.51	2.76 ± 0.50	2.75 ± 0.50	< 0.001	0.04
**Power plays (#)**	26.4 ± 17.7	18.5 ± 12.4	23.6 ± 12.6	< 0.001	0.06

Values are mean ± SD. sRPE-load: session rate of perceived exertion; PL/min: player load per minute.

* Training sessions, only

**FIG. 1:**
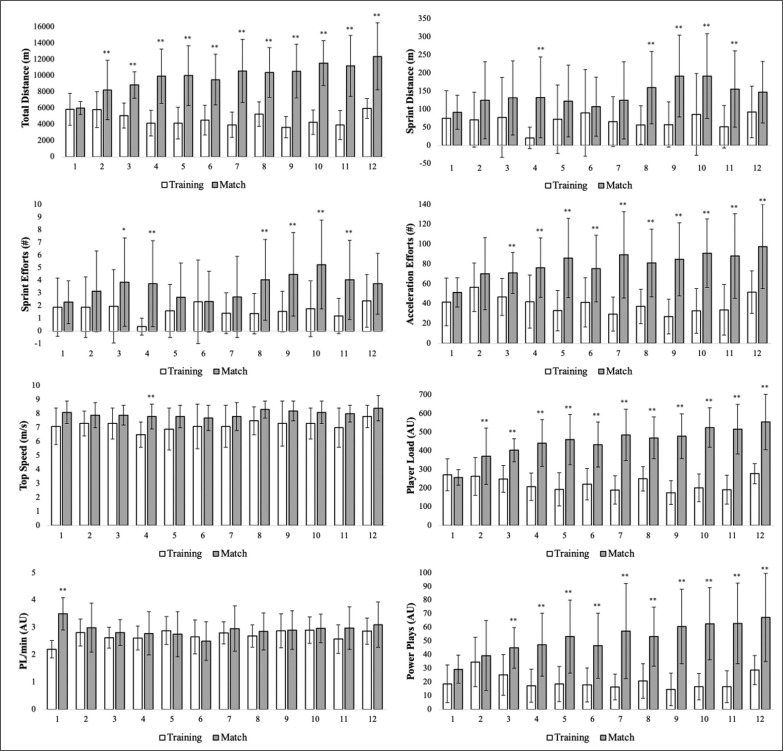
Weekly external load measures between training sessions and matches. * p < 0.01; ** p < 0.001; x-axis represents week.

Differences between weekly training session and match ELs are presented in Figure 1.

**FIG. 2:**
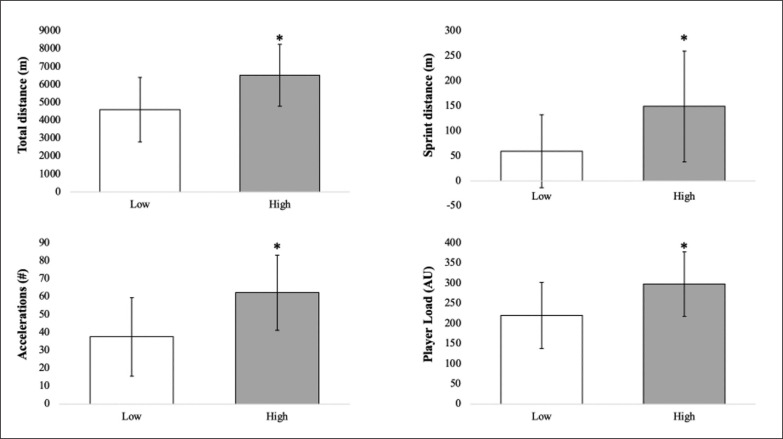
Training session total distance, player load, acceleration, sprint distance, and high-speed distance stratified by high (≥ 6) or low (≤ 5) perceived session exertion. * p < 0.001.

When training sessions were stratified and coded as high or low RPE, significant differences in total distance (high: 6531 ± 1731 m; low: 4608 ± 1809 m, p < 0.001), PL (high: 298 ± 80 AU; low: 221 ± 82 AU, p < 0.001), accelerations (high: 63 ± 21; low: 38 ± 22, p < 0.001), and sprint distance (high: 150 ± 111 m; low: 60 ± 73 m, p < 0.001) were observed (Figure 2).

**FIG. 3:**
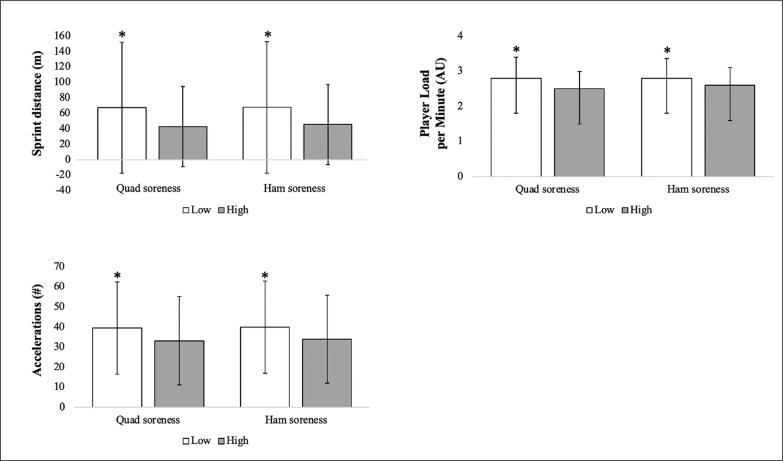
Training session acceleration, sprint distance, and PL/min stratified by high (≥ 3) or low (≤ 2) perceived quadricep and hamstring sorencess. * p < 0.001.

When training sessions were stratified and coded as high or low perceived quadricep and hamstring soreness, significant differences in same-day sprint distance (p = 0.006), acceleration (p = 0.02), and PL/min (p = 0.03) were observed (Figure 3).

Correlations coefficients, p-values, and 95% confidence intervals for all relationships can be found in Tables 3–5. sRPE-load was very strongly correlated with training session duration (r = 0.89, 95% CI [0.98, 0.99]), total distance (r = 0.88, 95% CI [0.89, 0.96]), accelerations (r = 0.73, 95% CI [0.67, 0.77]), top speed (r = 0.77, 95% CI [0.62, 0.79]), and PL (r = 0.88, 95% CI [0.85, 0.92]); strongly correlated with sprint distance (r = 0.56, 95% CI [0.55, 0.77]), and power plays (r = 0.67, 95% CI [0.76, 0.89]); and moderately correlated with PL/min (r = 0.43, 95% CI [0.29, 0.52]) (Table 3). Duration (t = 16.13, p < 0.001), total distance (t = 9.17, p < 0.001), sprint distance (t = 7.54, p < 0.001), PL (t = 4.22, p = 0.001), top speed (t = 4.69, p < 0.001), and acceleration (t = 2.02, p = 0.04) all significantly predicted post-training sRPE-load (F = 412.9, p < 0.001, R**^2^** = 0.75).

**TABLE 3:** Correlations between various external load measures and training session sRPE-load

Variable	r	95% CI	p-value
**Duration (min)**	0.89	0.98–0.99	< 0.001
**Total distance (m)**	0.88	0.89–0.96	< 0.001
**Sprint distance (m)**	0.56	0.55–0.77	< 0.001
**Accelerations (#)**	0.73	0.67–0.77	< 0.001
**Top speed (m/s)**	0.77	0.62–0.79	< 0.001
**Player load (AU)**	0.88	0.85–0.92	< 0.001
**PL/min (AU/min)**	0.43	0.29–0.52	< 0.001
**Power plays (#)**	0.67	0.76–0.89	< 0.001

PL/min: player load per minute

**TABLE 4:** Correlations between pre-training soreness and same-day workload measures

Quadricep Soreness				Hamstring Soreness			
Variable	r	95% CI	p-value	Variable	r	95% CI	p-value
**sRPE-load**	-0.01	-0.08 – 0.07	0.84	**sRPE-load**	-0.07	-0.13 – 0.03	0.07
**Duration**	0.02	-0.13 – 0.07	0.55	**Duration**	0.01	-0.10 – 0.09	0.72
**Total distance**	-0.12	-0.25 – -0.07	0.001	**Total distance**	-0.06	-0.17 – 0.01	0.08
**Sprint distance**	-0.17	-0.24 – -0.09	< 0.001	**Sprint distance**	-0.15	-0.25 – -0.09	< 0.001
**Sprint efforts**	-0.16	-0.26 – -0.08	< 0.001	**Sprint efforts**	-0.12	-0.25 – -0.07	0.001
**Accelerations**	-0.16	-0.24 – – 0.09	< 0.001	**Accelerations**	-0.11	-0.20 – -0.05	0.002
**Top speed**	-0.08	-0.47 – -0.08	0.04	**Top speed**	-0.07	-0.39 – 0.003	0.07
**Player load**	-0.13	-0.23 – -0.07	< 0.001	**Player load**	-0.08	-0.17 – -0.01	0.04
**PL/min**	-0.22	-1.21 – -0.57	< 0.001	**PL/min**	-0.14	-0.94 – -0.29	< 0.001
**Power plays**	-0.18	-0.31 – -0.11	< 0.001	**Power plays**	-0.10	-0.22 – -0.01	0.01

PL/min: player load per minute

**TABLE 5:** Correlations between workload measures and next-day soreness

Quadricep Soreness				Hamstring Soreness			
Variable	r	95% CI	p-value	Variable	r	95% CI	p-value
sRPE-load*	0.27	0.16–0.31	< 0.001	sRPE-load*	0.17	0.07–0.22	< 0.001
**Duration**	0.22	0.11–0.26	< 0.001	**Duration**	0.14	0.05–0.19	0.001
**Total distance**	0.18	0.10–0.25	< 0.001	**Total distance**	0.14	0.05–0.19	0.001
**Sprint distance**	0.04	0.04–0.12	0.29	**Sprint distance**	0.01	0.09–0.08	0.89
**Sprint efforts**	0.03	0.02–0.05	0.39	**Sprint efforts**	0.02	0.01– 0.03	0.89
**Accelerations**	0.09	0.01–0.16	0.04	**Accelerations**	0.08	0.002–0.15	0.06
**Top speed**	0.27	0.09–0.28	< 0.001	**Top speed**	0.17	0.03–0.21	< 0.001
**Player load**	0.16	0.09–0.24	< 0.001	**Player load**	0.13	0.04–0.19	0.003
**PL/min**	0.14	0.08–0.14	< 0.001	**PL/min**	0.13	0.06–0.15	0.003
**Power plays**	0.11	0.03–0.19	0.09	**Power plays**	0.11	0.01–0.18	0.01

PL/min: player load per minute.

* Training sessions, only

Pre-training quadricep soreness was weakly negatively correlated with total distance (r = -0.12, 95% CI [-0.25, -0.07]), sprint distance (r = -0.17, 95% CI [-0.24, -0.08]), power plays (r = -0.18, 95% CI [-0.31, -0.11]), sprint efforts (r = -0.16, 95% CI [-0.26, -0.08]), accelerations (r = -0.16, 95% CI [-0.24, – 0.09]), PL (r = -0.13, 95% CI [-0.23, -0.07]), and PL/min (r = -0.22, 95% CI [-1.21, -0.57]); and trivially correlated with top speed (r = -0.08, 95% CI [-0.47, -0.08]). Pre-training hamstring soreness was weakly negatively correlated with same-day workload measures, including sprint distance (r = -0.15, 95% CI [-0.25, -0.09]), power plays (r = -0.10, 95% CI [-0.22, -0.01]), sprint efforts (r = -0.12, 95% CI [-0.25, -0.07]), accelerations (r = -0.11, 95% CI [-0.20, -0.05]), and PL/min (r = -0.14, 95% CI [-0.94, -0.29]); and trivially correlated with top speed (r = -0.07, 95% CI [-0.39, 0.003])and PL (r = -0.08, 95% CI [-0.17, -0.01]) (Table 4).

sRPE-load (r = 0.27, 95% CI [0.16, 0.31]), session duration (r = 0.22, 95% CI [0.11, 0.26]), total distance (r = 0.18, 95% CI [0.10, 0.25]), power plays (r = 0.11, 95% CI [0.03, 0.19]), top speed (r = 0.27, 95% CI [0.09, 0.28]), PL (r = 0.16, 95% CI [0.09, 0.24]), and PL/min (r = 0.14, 95% CI [0.08, 0.14]) were weakly positively correlated with next-day quadricep soreness; sprint distance (r = 0.04, 95% CI [0.04, 0.12]), sprint efforts (r = 0.03, 95% CI [0.02, 0.05]), and acceleration efforts (r = 0.09, 95% CI [0.01, 0.16]) were trivially correlated. sRPE-load (r = 0.17, 95% CI [0.07, 0.22]), session duration (r = 0.14, 95% CI [0.05, 0.19]), total distance (r = 0.14, 95% CI [0.05, 0.19]), power plays (r = 0.11, 95% CI [0.01, 0.18]), top speed (r = 0.17, 95% CI [0.03, 0.21]), PL (r = 0.13, 95% CI [0.04, 0.19]), and PL/min (r = 0.13, 95% CI [0.06, 0.15]) were weakly correlated with next-day hamstring soreness (Table 5).

## DISCUSSION

Investigation of the relationships between ELs, sRPE-load, and perceived soreness throughout an entire NCAA DIII men’s collegiate soccer season is unique to the current study. The main findings include: 1) significant differences were observed in workloads across season phases, including notably high pre-season and post-season loads; 2) EL parameters significantly predicted (R**^2^** = 0.76) post-training sRPE-load; and 3) trivial and weak correlations existed between workloads and same-day and next-day self-perceived soreness measures.

Previous studies have demonstrated significant differences in workloads across season phases in collegiate [12] and professional [11] soccer populations, with pre-season consistently yielding greater total distance, high speed running, and sRPE-load than in-season [12]. Findings from the current study are in agreement, as higher pre-season workloads (training and match) were observed compared to in-season, although a seemingly higher total distance and lower sRPE-load than previously reported in NCAA DI men soccer athletes [12]. High pre-season workloads appear to be a consistent theme across NCAA DI and DIII men’s soccer [12, 21, 34], highlighting concerns over health, injury, and player availability. A congested pre-season phase (~2 weeks), characterized by steep workload intensification and accumulation, may be associated with maladaptations that can lead to injury/illness [1, 6, 8]. Thus, pre-season for collegiate soccer presents a heightened challenge characterized by two competing goals: 1) accumulating high physical and physiological workloads following the offseason period, and 2) managing workload accumulation in a manner that elicits positive adaptations, while avoiding maladaptive consequences prior to the start of the competitive season. These competing goals may be even more challenging to manage at the NCAA DIII level as athletic programs cannot mandate off-season team workouts or practices. Therefore, quantification of workloads is integral to the design of a productive pre-season period. Interestingly, the current study observed similar workloads between pre-season and post-season, which conflicts with prior research, which has found that post-season often reflects an intentional reduction of EL prescription as part of tapering strategies [12, 13]. One possible explanation is teams are often faced with more skilled opponents during the post-season phase, which may cause some teams to sustain greater ELs during post-season training in preparation for these competitions. Further, the time between the conclusion of the in-season phase and the first post-season match may differ between leagues and levels of play, which may preclude teams from implementing optimal tapering strategies. In the current study, athletes trained on three out of the four days between the final in-season and first post-season matches, with only one day off. Post-season tournament structure and match frequency may also influence the EL loads for various teams. Eight days separated the first and second post-season matches in the current study, possibly contributing to the increased workloads observed during this period. Nevertheless, this observation is novel and further investigation is warranted into the variance of ELs across season phases to optimize periodization schemes and match-play outcomes.

Of note, when training sessions were stratified based upon RPE (high: ≥ 6; low: ≤ 5), differences in ELs (total distance, PL, accelerations, and sprint distance) were evident. These findings are in alignment with prior research in NCAA DI women soccer players, signifying RPE is acutely sensitive to the changing magnitude of EL parameters across multiple levels, which supports the well-documented relationship between internal (e.g., RPE) and external (e.g., GPS) workload metrics [6, 14, 16, 21, 24] and further highlights the utility of RPE. Similarly, the current study observed differences in same-day sprint distance, acceleration, and PL/min when pre-training quadricep and hamstring soreness were stratified as high (≥ 3) or low (≤ 2). It appears intensity metrics are attenuated when athletes report high pre-session perceived soreness, pointing to the potential efficacy for coaching staffs to conduct soreness appraisals and implement an individualized approach to load-management strategies.

Training sRPE-load was positively correlated with session duration, total distance, PL, acceleration efforts, and top speed, with duration and total distance being significant predictors of sRPE-load, accounting for 73% of the variance. These results support previous findings that total distance is most strongly associated with IL [16, 35], pointing to the sensitivity of RPE to modulations in volume-load parameters. The strong relationship between total distance and sRPE-load has been reported in NCAA DI and DII men and women soccer players [6, 21, 36], professional men soccer players [17, 37], and youth soccer players [18, 35]. Further, similar relationships between PL and sRPE-load have been observed in professional [19] and semiprofessional [17] men soccer players, and NCAA DI women soccer players [6]. Of the aforementioned studies examining correlations within NCAA soccer players, only two [6, 36] demonstrated strong relationships between EL and sRPE throughout an entire season, meriting further investigation with season-long data. The very strong relationship between sRPE-load and volume metrics shows the utility of sRPE-load as a valid and reliable method of workload measurement. However, as sRPE-load is dependent upon total session duration, the relationship between volume-based parameters (i.e., total distance, PL) and sRPE-load, may be a reflection of session duration.

Previous investigations have demonstrated associations between changes in self-reported wellness measures and workloads during seasonal phases [21, 23] and entire soccer seasons [20, 22]. The current study uniquely examined these associations across a full NCAA DIII collegiate season (12 weeks) and discovered pre-training quadricep soreness was weakly correlated with same-day total distance, sprint distance, power plays, sprint efforts, accelerations, PL, and PL/min; and trivially correlated with top speed. Similarly, pre-training hamstring soreness was weakly correlated with sprint distance, power plays, sprint efforts, accelerations, and PL/min; and trivially correlated with top speed, and PL. Current results do not reflect the magnitude of correlation demonstrated in previous investigations that have reported a moderate to large negative correlation between same-day muscle soreness and sRPE-load [22], PL [23], total distance [23], and sprint distance [23] in professional men soccer players. However, data from Clemente (2018) were collected from small-sided games during the final two weeks of the season, which mirrors neither the season-long data collection period nor the diverse tactical structures and physiological demands of sessions as represented in the current study. Further, preseason morning soreness was found to negatively predict same-day total distance, PL, HSR, and sRPE-load in NCAA DI men soccer players [21]. However, the aforementioned studies employed a 7-point Likert scale assessing total body soreness, whereas the current study assessed specific muscle group (quadricep and hamstring) soreness, which may be partly responsible for the disparate outcomes. In addition, athletes in the current study submitted their soreness scales during the early afternoon period just prior to training, as opposed to a morning measurement. This distinction could explain the weak relationships observed between muscle soreness and sRPE-load, as afternoon scores may have been influenced by preceding physiotherapeutic treatment sessions and general movement/activity throughout the day. Thus, equivocal results appear to exist between perceived muscle soreness and same-day workload parameters across competition levels and durations of data collection periods, meriting further research into these relationships using a standardized approach to assessing soreness.

The current study investigated the relationship between EL, IL, and next-day soreness. In particular, sRPE-load, session duration, total distance, power plays, top speed, PL, and PL/min were weakly correlated with next-day quadricep soreness, while accelerations were trivially correlated with next-day quadricep soreness. Further, sRPE-load, session duration, total distance, power plays, top speed, PL, and PL/min were weakly correlated with next-day hamstring soreness. Taken together, these findings appear to support minimal associations between several workload metrics and next-day soreness. Previously, EL measures have been found to predict next-day perceptions of soreness and fatigue, respectively, including total distance, PL, HSR, and sRPE-load [21], while, IL measures (i.e., RPE) have been shown to demonstrate trivial to small correlations with next-day muscle status (i.e., muscle soreness) [38]. However, while wellness/soreness questionnaires – both standardized and customized versions – have been found to be valid measures of IL across a variety of populations [39, 40], challenges emerge when comparing results from different constructs and questionnaires across studies. Unlike the computation of IL via sRPE-load (i.e., RPE × session duration), which was designed for consistent implementation across studies [14, 15], the computation of IL via customized wellness questionnaires can yield variability. Specifically, the required movements, degree of contact and style of play of sports may influence the subsequent degrees of soreness experienced by athletes, which may influence the utility of certain wellness, or specifically soreness (i.e., whole-body versus muscle-specific) questionnaires. Alone, wellness constructs may be insufficient tools to quantify IL, but can play an important role in capturing the multifactorial nature of sport-induced physiological stress.

Although the first study to investigate the relationships between EL, sRPE-load, and perceived soreness throughout an entire NCAA DIII collegiate men’s soccer season, the current study is not without limitations. For example, only training sRPE-load and soreness measures were collected, thus, match-day data were not included in the analysis. Further, because quadriceps and hamstrings where the only muscles assessed for soreness, the lower extremity soreness metric used in the current study may not have captured the totality of perceived muscular soreness experienced by the athletes. Also, the use of different monitoring technologies, (i.e., video analysis, LPS, etc.) poses challenges to drawing comparisons across the literature, as each system may provide propriety metrics to classify match demands or exhibit varying degrees of accuracy, which may limit the generalizability to other programs.

## CONCLUSIONS

We found differences in workload measures between season phases in conjunction with differences in EL parameters when training session RPE and soreness were stratified into high and low categories. While significantly greater pre-season loads were observed compared to in-season, which supports previous results, the increased post-season loads relative to in-season is a novel finding. In accordance with previous investigations, the current study reported a very strong relationship between EL and sRPE-load, highlighting the utility of sRPE-load as a practical and affordable means of workload quantification, either alongside or in place of positional monitoring technology. Lastly, weak associations between workloads and soreness were observed. Due to conflicting results with prior research and the inherent difficulty in comparing results of wellness questionnaires across different studies and durations of data collection periods, more research is warranted into the relationship between soreness and workload and the efficacy of administering self-reported scales as primary quantifiers of IL. Nevertheless, soreness appraisals may provide coaches and practitioners with increased awareness of the cumulative physical “cost” of sport demands.
